# Patient-reported outcome (PRO)-based symptom assessment in patients with advanced lung cancer receiving first-line combination immunotherapy: a protocol for a multicenter, prospective, observational study

**DOI:** 10.1186/s12890-023-02432-5

**Published:** 2023-05-19

**Authors:** Yuanle Deng, Han Hu, Rong Jia, Wei Dai, Dengfeng Wang, Purong Zhang, Peng Zhang, Kai Cheng, Jianning Tang, Yan Wen, Xiang Zhou, Qiuling Shi, Zhujuan Xiong, Jin Zhou

**Affiliations:** 1 Department of Clinical Nutrition, Sichuan Clinical Research Center for Cancer, Sichuan Cancer Hospital & Institute, Sichuan Cancer Center, Affiliated Cancer Hospital of University of Electronic Science and Technology of China, Chengdu, China; 2 Department of Medical Oncology, Sichuan Clinical Research Center for Cancer, Sichuan Cancer Hospital & Institute, Sichuan Cancer Center, Affiliated Cancer Hospital of University of Electronic Science and Technology of China, Chengdu, China; 3 Department of Thoracic Surgery, Sichuan Clinical Research Center for Cancer, Sichuan Cancer Hospital & Institute, Sichuan Cancer Center, Affiliated Cancer Hospital of University of Electronic Science and Technology of China, Chengdu, China; 4 Gynecologic Oncology Center, Sichuan Clinical Research Center for Cancer, Sichuan Cancer Hospital & Institute, Sichuan Cancer Center, Affiliated Cancer Hospital of University of Electronic Science and Technology of China, Chengdu, China; 5 Department of Breast Surgery, Sichuan Clinical Research Center for Cancer, Sichuan Cancer Hospital & Institute, Sichuan Cancer Center, Affiliated Cancer Hospital of University of Electronic Science and Technology of China, Chengdu, China; 6 Department of Radiation Oncology, Radiation Oncology Key Laboratory of Sichuan Province, Sichuan Clinical Research Center for Cancer, Sichuan Cancer Hospital & Institute, Sichuan Cancer Center, Affiliated Cancer Hospital of University of Electronic Science and Technology of China, Chengdu, China; 7 Department of Pharmacy, Sichuan Clinical Research Center for Cancer, Sichuan Cancer Hospital & Institute, Sichuan Cancer Center, Affiliated Cancer Hospital of University of Electronic Science and Technology of China, Chengdu, China; 8 Department of Gastrointestinal Surgery, Sichuan Clinical Research Center for Cancer, Sichuan Cancer Hospital & Institute, Sichuan Cancer Center, Affiliated Cancer Hospital of University of Electronic Science and Technology of China, Chengdu, China; 9 School of Public Health, Chongqing Medical University, Chongqing, China

**Keywords:** Patient-reported outcome, Lung cancer, Immunotherapy, Immune-related adverse events

## Abstract

**Background:**

Immunotherapy is currently applied in the first-line treatment regimens for numerous advanced cancers, especially advanced lung cancer. Immune-related adverse events (irAEs) resulting from immunotherapy can vary in severity and cause a substantial symptom burden to patients. However, there are limited data on symptom burden in patients with advanced lung cancer following immunotherapy. To address this deficit, this study aims to provide insight into the symptom burden and severity through patient-reported outcome measurements and conduct an analysis of temporal trends and clinical consequences of symptom burden in patients with advanced lung cancer receiving combination immunotherapy.

**Methods:**

We will prospectively recruit 168 eligible patients from 14 hospitals in China. Eligible patients will be aged ≥ 18 years, pathologically diagnosed with locally advanced or stage IV primary lung cancer without surgical indications, and agreed to receive immunotherapy in combination with other therapies. The primary outcome of this study is the symptom burden of patients during the immunotherapy course. Longitudinal symptom data will be collected using the MD Anderson Symptom Inventory–Lung Cancer module (MDASI-LC) and the symptomatic irAEs scale at baseline (once before treatment) and weekly after treatment, until 1 month after the last treatment cycle has been completed. The trajectory of symptom burden following combination immunotherapy will be depicted, and by linking it to clinical outcomes (the secondary outcome and exploratory outcome of this study), the consequence of symptom burden in patients with advanced lung cancer receiving combination immunotherapy will be examined further.

**Discussion:**

This study intends to establish longitudinal symptom trajectories in patients with lung cancer receiving immunotherapy, and explore its association with clinical outcomes. These findings may serve as an important reference for clinicians in the symptomatic management of patients with lung cancer receiving immunotherapy.

**Trial registration number:**

ChiCTR2200061540. Registered on June 28, 2022.

## Background

Lung cancer is the second most common malignancy and most lethal cancer worldwide, with highest morbidity and mortality rates in China [1]. Many patients with lung cancer are already in the advanced stages at initial clinical diagnosis, thereby having poor prognosis and a relatively short overall survival. Cancer immunotherapy, especially immune checkpoint inhibitors (ICIs), is an emerging therapeutic strategy, and combination therapy with traditional chemotherapy or radiation therapy has played an important role in the treatment of advanced lung cancer. It has markedly improved the survival of some patients, and a significant fraction of patients can achieve long-term survival of ≥ 5 years [2]. Therefore, regarding ICIs, the quality of life of patients with long-term survival is extremely important. Combination immunotherapy for lung cancer is associated with significant treatment-related symptoms [3]. Therein, irAEs following ICIs generally have a long duration and broad spectrum [4] and can cause physical and psychosocial burdens on patients, thereby negatively affecting quality of life and compliance with therapy.

Symptoms in patients with lung cancer receiving immunotherapy are traditionally assessed by the medical staff rather than self-reports from patients [5, 6]. PRO (patient-reported outcome) is a non-traditional clinical indicator of capturing patient symptoms, functional outcomes, or health-related quality of life directly from the patients’ perspective without interpretation from others [7, 8]. Screening of PRO followed by tailored interventions can achieve better clinical outcomes [9]. In patients with advanced lung cancer, much attention has been focused on tracking adverse event symptoms induced by chemotherapy, which showed significant survival benefits when combined with threshold-driven alter and response [10, 11].

However, the longitudinal symptom trajectories after immunotherapy treatment in patients with advanced lung cancer have not been addressed yet. Particularly, immunotherapy is a new treatment paradigm and can lead to complex irAEs with relative specificity. Addition of PROs to traditionally collected outcomes can provide a comprehensive overview of patient experience during and after treatment [9]. It is, therefore, necessary to track the symptom trajectories after immunotherapy treatment directly from patient self-reports. Moreover, the current understanding of the association between patient-reported symptoms following immunotherapy and clinical outcomes is limited, and only a few indicators (e.g., overall survival and progression-free survival) have been reported [12]. The connection between symptom burden and clinical outcomes, including the completion rate of immunotherapy, timeliness of treatment, complication rate, and unplanned clinic rate may be critical for clinicians to manage adverse events.

However, some questions and challenges remain unaddressed for symptom assessments in patients with lung cancer receiving immunotherapy. First, immunotherapy-specific items are noticeably missing in all lung cancer modules of commonly used PRO measures (PROM). For instance, although the MDASI-LC is widely used in the medical community at present, it is primarily designed for patients receiving chemoradiation [13], and is applied in the realm of radiotherapy [14] and operation [15]. Some frequently reported irAEs symptoms, such as diarrhea, rash, and fever, have not been included, limiting its application in detecting patients’ experience during immunotherapy. Therefore, updating those measures by introducing immune-specific PRO symptom clusters for better application in both clinical practice and research is essential. Second, optimal symptom monitoring time points for immunotherapy remain undefined, while irAEs are characterized by recurrent, long-standing, and dynamic changes as well as sudden and dramatic adverse prognostic factors [16]. The time points of PRO data collected during hospitalization and after discharge should be combined with the above characteristics to avoid the omission of real symptoms and artifacts and reflect the rules of immunotherapy, with the consideration of system burden-induced by frequent assessment schedule. Third, warning signals of irAEs for medical intervention require more evidence for support. In clinical practice, irAEs with complexity and relative specificity are difficult to be precisely managed. Thorough insight into the connection between irAEs and key clinical outcomes will help in clinical decision-making and management.

Thus, using a multicenter longitudinal cohort design, this study aims to leverage PRO data to establish temporal trends in the symptom burden of patients with advanced lung cancer receiving combination immunotherapy, and examine its association with clinical outcomes. These findings are necessary for clinical decision-making and supportive care for patients with advanced lung cancer receiving immunotherapy.

## Methods/Design

### Study design

**Fig. 1:**
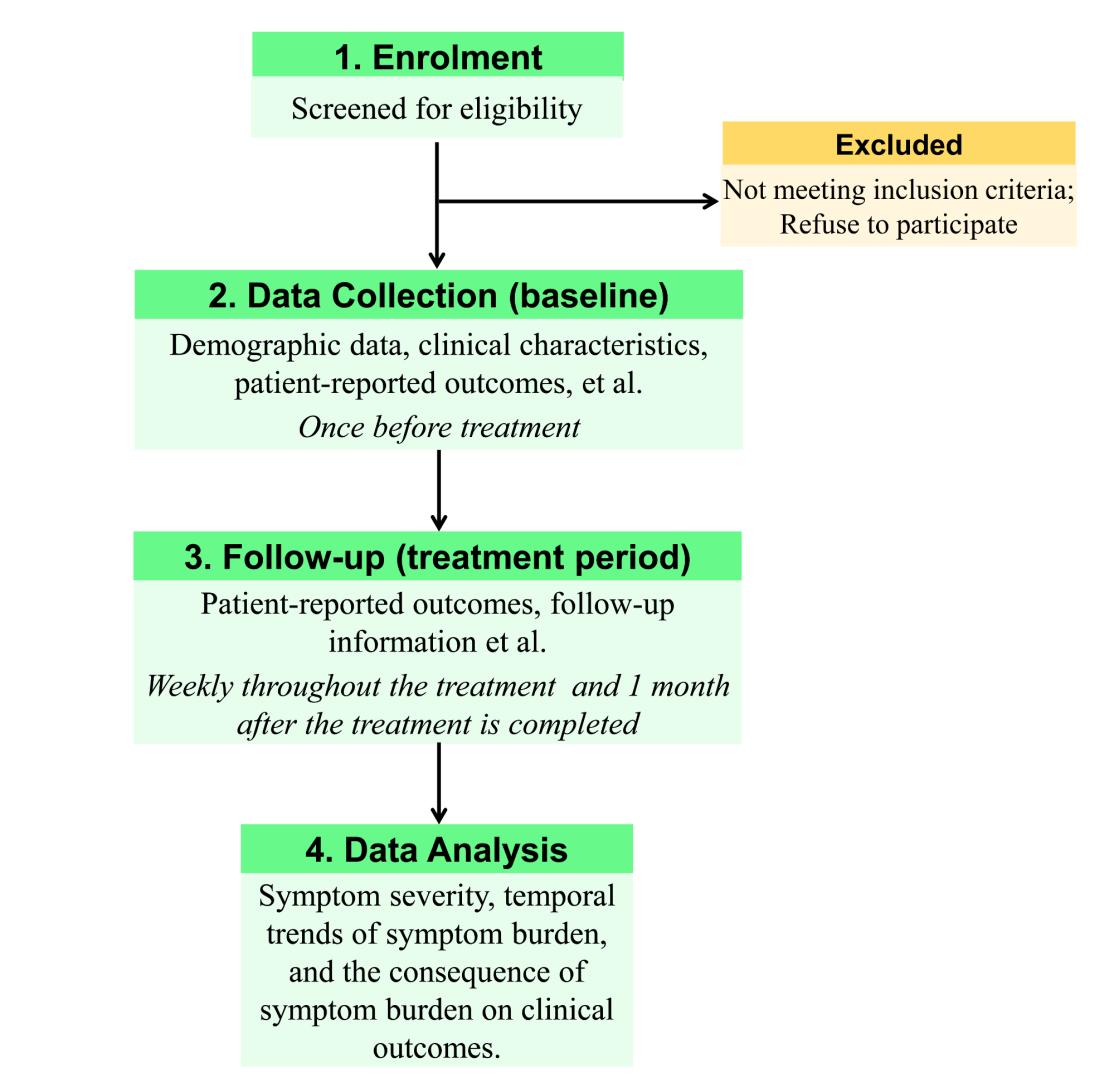
The flow chart of this study

This is a multicenter observational prospective cohort study. The flowchart of the study is shown in Fig. 1.

### Study setting

The study will be initiated by Sichuan Cancer Hospital and conducted in 14 hospitals in China, namely, Sichuan Cancer Hospital, Third People’s Hospital of Chengdu, Seventh People’s Hospital of Chengdu, Western Theater General Hospital, Sichuan Provincial People’s Hospital, First Affiliated Hospital of Chengdu Medical College, Chengdu Second People’s Hospital, Mianyang Central Hospital, Nanchong Central Hospital, West China Hospital of Sichuan University, Deyang people’s Hospital, Suining people’s Hospital, Dujiangyan people’s Hospital and Chengdu Fifth People’s hospital. Each patient will be followed up from before the treatment to 1 month after the completion of the last cycle of immunotherapy. This study has been ongoing since June 2022 and is currently planned to be continued until December 2024.

### Study population

The patients included in the study are those with a diagnosis of advanced primary lung cancer and prescribed combination immunotherapy as first-line treatment. The inclusion and exclusion criteria for this study are listed in Table 1.

**Table 1:** The inclusion and exclusion criteria for this study

Inclusion criteria	Exclusion criteria
✓ Pathologically confirmed advanced or stage IV primary lung cancer who had no surgical indicators.	× Age < 18 years old at the time of informed consent.
✓ Scheduled to receive combination immunotherapy as the first-line treatment.	× Cognitive impairment or inability to understand the research content.

### Withdrawal criteria

Participants will be withdrawn from the study, and no further data will be collected if they meet the criteria, as shown in Table 2.

**Table 2:** The withdrawal criteria for the study

Withdrawal criteria
1. Cancelling planned immunotherapy before the treatment.
2. Patients who break the study protocol (deliberately offering inaccurate PRO data).
3. Patients who demand an immediate withdraw from the research.
4. Severe complications interfering with PRO data collection.
5. Other conditions that require withdrawal as assessed by the investigator.

### Sample size calculation

This study will mainly focus on establishing temporal trends in the symptom burden of patients with advanced lung cancer receiving combination immunotherapy and symptom burden in patients with adverse events should be distinguished from that in patients without adverse events. It is estimated that adverse events develop in 30% of patients receiving immunotherapy, and our preliminary research data showed that the symptom score of the patients with adverse events was 1.6 ± 1.1, and the symptom score of the patients without adverse events was 1.0 ± 0.7. According to the t-test of the two groups’ mean and 20% of dropout rate, with a type I error rate of 5% and a power of 90%, using two-sided test, the final sample size is approximately 168 cases.

### Outcome measures

#### Primary outcome

The primary outcome of this study is the symptom burden in patients with lung cancer during the first-line combination immunotherapy course. It will be assessed using the MDASI-LC and symptomatic irAEs scale directly from patient self-reports. MDASI-LC [17], the lung cancer-specific PRO measurement, will be translated into simplified Chinese to collect longitudinal symptom data once before treatment (typically within 3 days before treatment) and weekly throughout the treatment and till 1 month after treatment completion. It comprises 16 symptoms (pain, fatigue, nausea, disturbed sleep, distress, short breath, remember things, lack of appetite, drowsy, dry mouth, sad, vomiting, numbness, cough, constipation, and sore throat) and six functional items (activity, mood, work, relations, walking, and enjoy life). Each item uses a 0-10-point scale, with 0 representing “symptom has not been present or no functional impairment” and 10 representing “the most severe symptoms or functional impairment”. In addition, the symptomatic irAE scale is a subgroup of symptomatic irAE items from the Patient Reported Outcomes version of the Common Terminology Criteria for Adverse Events library [18, 19], which provides a valid and reliable assessment of symptomatic toxic effects from the patient’s perspective, and is encouraged for use in oncology trials to enhance the accuracy of adverse event reporting [19]. The scale consisting of 15 items (bellyache, diarrhea, bloated pain, night sweats, headache, rash, chills and fever, pruritus, pectoralgia, hemoptysis, abdominal, early satiety, taste change, smell change, and palpitation) will be translated into Chinese to collect the severity score of irAEs. Like the MDASI-LC, it also uses a 0–10 scale, where 0 represents “symptom has not been present or no functional impairment” and 10 represents “the most severe symptoms or functional impairment”.

#### Secondary outcome

The secondary outcomes will include the incidence rate of grade 3–4 immunotoxicity, the completion rate of six cycles of immunotherapy, the timeliness of treatment, complication rate, and unplanned clinic rate. Immunotoxicity will be assessed based on the Common Terminology Criteria for Adverse Events (CTCAE v.5) criteria, and irAEs will be classified according to the affected system, including respiratory, gastrointestinal dermatological, hepatic, articular, neurological, and endocrine systems.

#### Exploratory outcome

The exploratory outcomes will include overall survival (OS) and progression-free survival (PFS). PFS and OS are defined from start of treatment until recurrence or date of death.

### Data collection, management, and monitoring

**Fig. 2:**
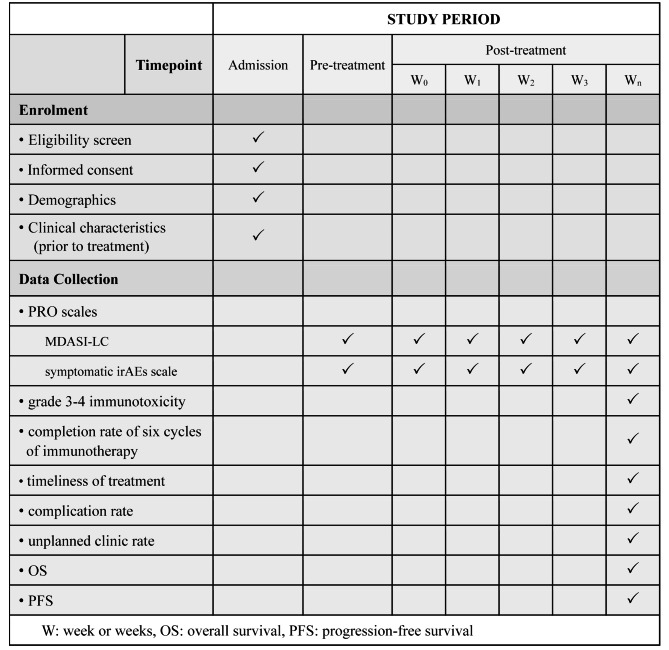
Study schedule of enrolment, data collection and timeline

The collected data will include demographics, clinical characteristics before treatment, PROs, and clinical outcomes as mentioned above. PRO data will be collected at baseline (before treatment), treatment onset, and once a week (± 2 days) after receiving treatment until admission to the hospital for the next cycle of treatment. Furthermore, the previous step of PRO data collection will be repeated until six cycles of immunotherapy have completed for 1 month. The study schedule of this study is shown in Fig. 2.

An electronic questionnaire is the primary form of PROs data collection, which will be sent to patients through a link or quick response code and will be completed by the patients on their own unless they ask for assistance. In addition, demographics and clinical characteristics before treatment will be obtained from the electronic medical record system, and other data will be obtained through telephone follow-up.

The project team is composed of multiple functional roles, including a data manager, a data monitor, and data quality control staff. The data manager is responsible for organizing the form design, database establishment, standard operating procedure (SOP) production, researcher training, and daily management. REDCap, a web-based software application for data storage and management, will be utilized for case report form design, database establishment, and data management in our study. Regarding the data monitor and quality control staff, the data monitor will take charge of inspecting the entered data once every 3 months, and the quality control staff will be responsible for the quality of the data and the progress of the study.

### Quality control

The present study will be implemented by and receive guidance and supervision from a multidisciplinary team comprising the principal investigator, subcenter principal, clinicians, clinical research methodologists, statisticians, research assistants, data managers, data monitors, and data quality control staff. A SOP will be proposed as an important reference to guarantee the quality of entered data, and all participants involved in the study are bound to receive SOP training prior to starting the recruitment of patients. As for subcenters, investigators will receive regular online guidance and telephone supervision, and on-site monitoring from the principal investigator to control the quality of data entry. When entering the data, the REDCap system can immediately execute automatic logical verification to correct the faults in this process. In addition, there will be regular and non-scheduled data checking during the patient recruitment process, and all data will receive a final verification for completeness and accuracy after data entry.

### Data confidentiality

To protect data privacy, participants involved in the study will get a unique study ID (consisting of two sets of numbers) for personal identifying information that is not irrelevant to the assessment. All data (including all personal data) will be stored in the REDCap of Sichuan Cancer Hospital, and all investigators will have access to them.

### Data analysis

Participants who meet the withdrawal criteria for the study will not be included in the final analysis. For the study results, descriptive analysis will be undertaken by using mean ± SD or median with interquartile range (IQR) for continuous variables, and absolute number (n) and proportion (%) for categorical variables. First, we will use descriptive statistics to describe the severity and time trend of symptom burden and functional interference in patients treated with combination immunotherapy. Symptom trajectories will be depicted with line graphs describing the severity score of patients reporting symptoms from pre-treatment to complete the whole treatment course for 1 month. Furthermore, the association between symptom and clinical outcomes will be examined. Specifically, the symptoms will be categorized based on the severity, and the difference in clinical outcomes (including incidence rate of grade 3–4 immunotoxicity, the completion rate of six cycles of immunotherapy, the timeliness of treatment, complication rate, unplanned clinic rate, OS and PFS) will be assessed. We will use R v.4.2.0 to perform the statistical analysis, and differences will be considered statistically significant if the two-tailed *p*-values are < 0.05.

## Discussion

Cancer and its treatments often cause multiple symptoms in patients, negatively influencing their prognosis and contributing to physical and psychological consequences [20]. Treatment-related adverse events severely affect prognosis in most cases, and the link between irAEs and prognosis is complicated and related [21]. Although irAEs with delayed onset and prolonged duration are easily neglected [22], organ injury or death resulting from irAEs can have a detrimental effect on patient survival [23], and permanent ICIs cessation due to irAEs can adversely affect treatment benefits [24]. It is therefore evident that symptoms of patients with cancer after treatment with immunotherapy need to be followed up effectively and completely. Electronic patient-reported outcomes (ePROs), which comprise health-related questionnaires completed by the patient themselves, are a promising approach for follow-up and can effectively detect symptoms and their severity. Additionally, as a new follow-up model, it has been reported to be timely and cost-effective and employs a longitudinal collection of patient-symptom data [25] .

Therefore, this study is focused on the assessment of the symptoms following combination immunotherapy, through the application of the ePRO model. Potential implications of the findings include [1] identifying the targeted symptoms for patients with advanced lung cancer receiving first-line combination immunotherapy; [2] demonstrating the longitudinal symptom trajectories of first-line combination immunotherapy for lung cancer; and [3] examining the correlation between symptom burden and clinical outcomes (including incidence rate of grade 3–4 immunotoxicity, the completion rate of six cycles of immunotherapy, the timeliness of treatment, complication rate, unplanned clinic rate, OS and PFS).

ePROs are a novel and significant way to clinically follow up symptoms after treatment with immunotherapy by focusing on patients’ views and experiences. Using this method, this study will help understand symptom trajectories following combination immunotherapy, which is important in improving preparedness for immunotherapy treatment as well as setting up individual strategies to optimize patient experience and outcomes.

## Trial status

Patient recruitment commenced in July 2022 and is currently in progress. This study is planned to be conducted until December 2024.

## Data Availability

No additional data and materials available.

## Declarations

### Patient and public involvement statement

Patients will be invited to participate in the study design, patient recruitment, and study implementation. The general public will not be invited to participate in the study design, recruitment, and implementation. Patients will not be informed of the results of the study unless they request it at the time of enrolment.

## List of Abbreviations

CTCAE: Common Terminology Criteria for Adverse Events
ePROs: Electronic patient-reported outcomes
irAEs: Immune-related adverse events
ICIs: Immune checkpoint inhibitors
MDASI-LC: MD Anderson Symptom Inventory–Lung Cancer module
OS: Overall survival
PFS: Progression-free survival
PRO: Patient-reported outcome
PROM: patient-reported outcomes measures
SOP: Standard operating procedure
